# Genome-scale modeling using flux ratio constraints to enable metabolic engineering of clostridial metabolism *in silico*

**DOI:** 10.1186/1752-0509-6-42

**Published:** 2012-05-14

**Authors:** Michael J McAnulty, Jiun Y Yen, Benjamin G Freedman, Ryan S Senger

**Affiliations:** 1 Biological Systems Engineering Department, Virginia Tech, Blacksburg, VA 24061, USA

**Keywords:** Genome-scale model, clostridia, flux ratio, flux balance analysis, metabolic engineering, systems biology

## Abstract

**Background:**

Genome-scale metabolic networks and flux models are an effective platform for linking an organism genotype to its phenotype. However, few modeling approaches offer predictive capabilities to evaluate potential metabolic engineering strategies *in silico*.

**Results:**

A new method called “flux balance analysis with flux ratios (FBrAtio)” was developed in this research and applied to a new genome-scale model of *Clostridium acetobutylicum* ATCC 824 (*i*CAC490) that contains 707 metabolites and 794 reactions. FBrAtio was used to model wild-type metabolism and metabolically engineered strains of *C. acetobutylicum* where only flux ratio constraints and thermodynamic reversibility of reactions were required. The FBrAtio approach allowed solutions to be found through standard linear programming. Five flux ratio constraints were required to achieve a qualitative picture of wild-type metabolism for *C. acetobutylicum* for the production of: (i) acetate, (ii) lactate, (iii) butyrate, (iv) acetone, (v) butanol, (vi) ethanol, (vii) CO_2_ and (viii) H_2_. Results of this simulation study coincide with published experimental results and show the knockdown of the acetoacetyl-CoA transferase increases butanol to acetone selectivity, while the simultaneous over-expression of the aldehyde/alcohol dehydrogenase greatly increases ethanol production.

**Conclusions:**

FBrAtio is a promising new method for constraining genome-scale models using internal flux ratios. The method was effective for modeling wild-type and engineered strains of *C. acetobutylicum*.

## Background

### Modeling clostridial metabolism

Butanol is of considerable research interest as a potential biofuel, and its renewable production through fermentation is sought largely from the clostridia. In particular, *Clostridium acetobutylicum* ATCC 824 has been one of multiple clostridia researched for butanol production over the past few decades. In fact, the first applications of metabolic flux balancing were performed using a model of *C. acetobutylicum* primary metabolism to understand what caused this organism to produce butanol and the competing metabolic byproducts: (i) acetate, (ii) butyrate, (iii) lactate, (iv) acetone, (v) ethanol, and several others in small amounts [1,2]. Flux modeling of the primary metabolism of *C. acetobutylicum* has led to a better understanding of the role cofactor balancing plays in directing global metabolic changes. It has played a significant role in metabolic engineering by identifying bottlenecks and critical flux distributions at metabolic branch points [3-8]. Multiple “genome-scale” metabolic network reconstructions now exist for *C. acetobutylicum*[9-12]. Similar networks and their corresponding genome-scale models have been reviewed extensively [10,13-20]. In general, they are used to (i) complete genome annotation [21], (ii) predict optimal culturing conditions [22-24], (iii) discover genomic regulation [20,25,26], (iv) identify essential genes and drug targets [27-33], (v) study strain evolution [34,35], and (vi) design productive strains [36-38]. Modeling results on the genome-scale have been applied to both “acidogenic” and “solventogenic” programs of clostridial metabolism [9]. The acidogenic program is characterized by high acids (i.e., acetate and butyrate) production and high growth rates, and the solventogenic program (i.e., acetone and butanol production) largely coincides with the stationary growth phase of the culture. During solventogenesis, acetate and butyrate are re-consumed by the culture and converted to acetone and butanol. The genetic program of this metabolic shift between acids and solvents production has been studied in detail [39]. Several insights into *C. acetobutylicum* metabolism have been gained from “gap filling” the metabolic network by locating previously unknown enzymes and biochemical reactions [11,12]. The total rate at which a cell produces/consumes protons through the several membrane transport mechanisms is termed the specific proton flux (SPF), and this parameter has shown to significantly reduce the total number of flux “solutions” available for the under-determined genome-scale model of *C. acetobutylicum*[12]. Reducing the number of degrees of freedom of these genome-scale models through application of genetic regulation and physicochemical constraints has been recognized as a key strategy for generating metabolic flux predictions that coincide with experimental observations [20].

### Engineering clostridial metabolism

**Figure 1:**
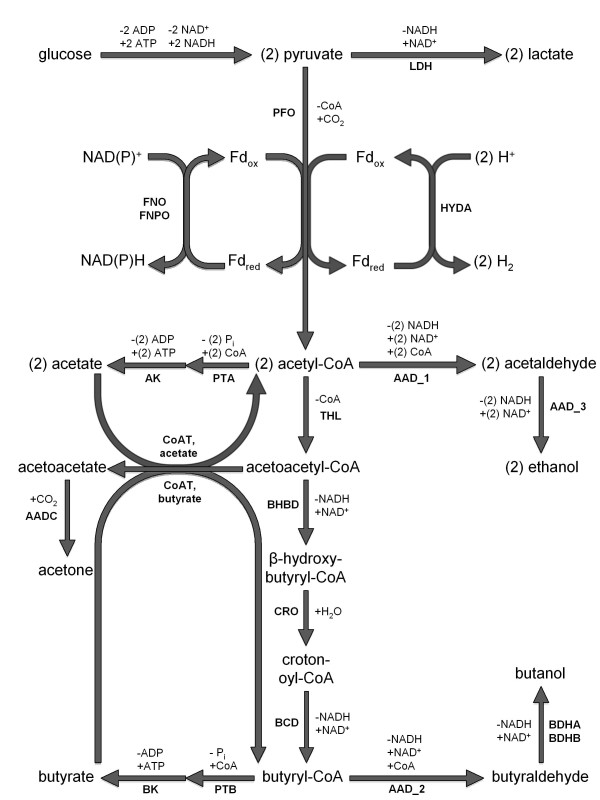
**Primary central carbon metabolism of *C. acetobutylicum.*** Cofactors consumed by each reaction are listed as (−) and cofactors produced (+) (H^+^ ions are not shown). The following enzymes are shown in bold: (LDH) lactate dehydrogenase, (PFO) pyruvate ferredoxin oxidoreductase, (FNO) ferredoxin NAD^+^ oxidoreductase, (FNPO) ferredoxin NADP^+^ oxidoreductase, (HYDA) hydrogenase, (AAD) acetaldehyde/alcohol dehydrogenase, (PTA) phosphotransacetylase, (AK) acetate kinase, (THL) thiolase, (CoAT) acetoacetyl-CoA transferase (for acetate and butyrate), (AADC) acetoacetate decarboxylase, (BHBD) β-hydroxybutyryl-CoA dehydrogenase, (CRO) crotonase, (BCD) butyryl-CoA dehydrogenase, (PTB) phosphotransbutyrylase, (BK) butyrate kinase, (BDHA) butanol dehydrogenase A, and (BDHB) butanol dehydrogenase B. The CoAT can function with either acetate or butyrate substrate; it does not require both. The AAD can catalyze three reactions in the model. These are listed as (i) AAD_1, (ii) AAD_2, and (iii) AAD_3.

Knowledge of the metabolic pathways of butanol fermentation has allowed for targeted engineering approaches. As acids (acetate and butyrate) and alcohols (ethanol and butanol) are competing products of fermentation, metabolic engineering strategies have been designed to silence acid producing pathways in attempt to re-direct carbon flow into the alcohol producing pathways. The primary metabolic network of *C. acetobutylicum* is shown in Figure 1 (adapted from [6,8]). An example of this strategy is the knockdown of the butyrate kinase (*buk*) (BK in Figure 1) and phosphotransacetylase (*pta*) (PTA in Figure 1) genes in clostridial metabolism [40]. The proximity of the *pta* gene in the genome to the acetate kinase (*ak*) (AK in Figure 1) gene resulted in the silencing of both genes simultaneously, further decreasing acetate production. The results of this engineering strategy showed that eliminating genes of the acetate pathway had little effect compared to the wild-type, while knockdown of butyrate pathway genes resulted in 10% more butanol and 50% less acetone than the wild-type [41]. The aldehyde/alcohol dehydrogenase gene (*aad* or *adhE1*) (AAD in Figure 1) was over-expressed in the presence of a *buk* knockout, and this strain yielded a 300% increase in butanol production and 400% increase in ethanol production over the wild-type strain [42]. In a separate metabolic engineering strategy, the *aad* was over-expressed while knocking-down the gene for subunit B of acetoacetyl-CoA transferase (*ctfB*) (CoAT in Figure 1). This led to a strain with similar butanol productivity but the ability to produce extraordinary ethanol concentrations of 200 mM (23-fold higher than the wild-type) [43]. However, when the *aad* gene was put under control of the *ptb* gene promoter (to increase expression during the early acidogenic phase of the culture) an increase in butanol concentrations to 300 mM (a record high) was observed along with faster accumulation of butanol in the culture [44].

### Metabolic engineering *in silico*

The goal of metabolic engineering *in silico* is to derive (or at least evaluate) potential metabolic engineering strategies prior to constructing them in the laboratory. For example, will a particular gene over-expression or knockout in *C. acetobutylicum* increase butanol production? Answering questions of this type is one of the potential uses of genome-scale modeling. However, with the initial genome-scale model for *C. acetobutylicum*[11,12], these questions could not be addressed without constraints on acid/solvent production. These constraints artificially specified ranges for secretion rates of acid and solvent products. These were necessary due to the large number of degrees of freedom that exist in the under-determined genome-scale model and the high degree of branching in the primary metabolism of clostridia. Simply, too many flux solutions were available if the user was only to define the substrate uptake rate and a proper objective function. The production of products/byproducts by a metabolic network not only completes elemental balances but it also regenerates and balances cofactors. In clostridial metabolism, ATP is regenerated by the production of acetate or butyrate, and NAD^+^ is produced by the production of (i) lactate, (ii) ethanol, or (iii) butanol. With several options to balance cofactors available, information about enzyme specificity is necessary to achieve reasonable selectivity. If constraints in a genome-scale model are simply placed around secretion of a product or byproduct, the model does not represent the cellular mechanisms that result in proper selection. Thus, an effective metabolic engineering strategy cannot be formulated *in silico* given these types of constraints.

With the ultimate goal of re-directing metabolic flux through the butanol production pathway in *C. acetobutylicum*, few tools, with the notable exception of OptKnock [36], exist for deriving a metabolic engineering strategy. Even with its many successes, OptKnock is restricted to gene knockouts and cannot suggest over-expression and partial gene knockdown strategies to engineer metabolism. However, the recently published OptForce algorithm [38] provides the capability to identify both gene over-expressions and knockdowns required of a metabolic network to produce a targeted amount of a specified product. Ultimately, methods that target the regulatory network of the cell and re-direct metabolic flux at network branch points will enable even more effective metabolic engineering *in silico.* The research presented here is a first step to constraining metabolic branching based on enzyme specificity. This approach also enables simulation of gene over-expressions and partial gene knockdowns in addition to gene knockouts.

### Considering metabolic flux ratios

The experimental determination of metabolic flux and pathway usage through the use of isotope tracers has significantly contributed to the overall understanding of regulated metabolism. One approach to characterize metabolism is through the use of metabolic flux ratio analysis (METAFoR) [45-47]. This method is used to determine the degree of converging pathway usage to produce a metabolite pool when multiple synthesis routes exist. For example, METAFoR can reveal the relative contributions of anaplerosis and the TCA cycle to the formation of the oxaloacetate pool. Early results revealed the robustness of central carbon metabolism of *Escherichia coli*[45,47] as many calculated flux ratios were found impervious to genetic perturbations. Additional computational method development led to the formulation of constraints for flux balancing from measured flux ratios [48,49]. The resulting algorithm was effective given small metabolic networks of primary metabolism and the use of nonlinear programming methods. Unfortunately, these aspects have limited the applicability to large genome-scale metabolic networks, which often must rely on linear programming.

### Genome-scale modeling with flux ratios

Of the several successful (and unsuccessful) metabolic engineering strategies applied to clostridia (many of which are not mentioned here), it was not immediately apparent which design(s) would be successful upon conception. The mutant strains had to be created in the laboratory and analyzed. From these results, hypotheses were formed that guided more advanced designs. The purpose of metabolic engineering *in silico* is to analyze and optimize engineering strategies *a priori* so that only the most promising candidates are constructed in the laboratory. While genome-scale modeling has provided the necessary platform for metabolic engineering *in silico*, the large number of degrees of freedom of these models has been limiting. Here, a new approach called “flux balance analysis with flux ratios (FBrAtio)” is developed and applied. One significant advantage of FBrAtio is that flux ratio constraints are built into the stoichiometric matrix directly. This approach allows for multiple flux ratio constraints to be included simultaneously, and the flux balancing problem can be solved using simple linear programming. In particular, FBrAtio is used to show that the butanol to acetone production ratio of *C. acetobutylicum* increases in the presence of CoAT knockdown by antisense RNA (asRNA). This metabolic engineering strategy is also simulated in the presence of AAD knockdown and over-expression to show this method can predict these published outcomes [43,50].

## Methods

### Genome-scale model

A new genome-scale model for *C. acetobutylicum* ATCC 824 was constructed by expanding the previously published model by Senger and Papoutsakis [11,12]. The new model is called *i*CAC490 and contains 707 metabolites involved in 794 biochemical reactions, including 66 membrane transport reactions. The model includes 490 genes from the *C. acetobutylicum* genome. The newly updated *i*CAC490 model differs from the original Senger and Papoutsakis model [11,12] in that it contains 242 more reactions (a 44% increase) and 285 more metabolites (a 68% increase). The new reactions added to create the *i*CAC794 model were obtained from the KEGG database [51] and recent literature. The *i*CAC490 model also contains an updated TCA cycle that operates in both oxidative and reductive directions to succinate, as shown by recent fluxomics studies [52,53]. The model allows the export of succinate since its metabolic fate has not yet been resolved conclusively. The *i*CAC490 model is also fully compartmentalized and allows the presence of chemical reactions in the extracellular environment. Thermodynamic reaction reversibility constraints based on Gibb’s free energy calculations from the group contribution method [54,55] have also been applied. The biomass equation was also updated for the *i*CAC490 model, using the initial version by Senger and Papoutsakis [11,12] as a template. A nonlinear optimization procedure was applied (manuscript in preparation) to optimize the biomass equation given specific environmental conditions. The biomass equation derived for exponential growth was used extensively in simulation studies reported here. It was found that the exponential growth biomass equation could result in qualitatively accurate model predictions. It is acknowledged that an updated and dynamic biomass equation will be required to obtain model predictions that are quantitatively accurate. The reconstructed metabolic network of the *i*CAC490 model is included as Additional file 1. The SBML formatted model is included as Additional file 2.

### Flux balance analysis

The *i*CAC490 genome-scale model was simulated using flux balance analysis through the COBRA toolbox [56]. The open-source GLPK linear programming software was used to solve the flux balance equation (*S · v* = 0), where *S* is a stoichiometric coefficient matrix and *v* is a vector of flux values. Methods related to construction of the stoichiometric matrix and the required steady-state approximation for intracellular metabolite concentrations have been detailed elsewhere [57]. The objective functions used for all simulations were (i) maximizing the specific growth rate of the cell while (ii) minimizing the total flux of the system.

### The specific proton flux

The concept of the specific proton flux (SPF) was first introduced by Senger and Papoutsakis [12] and describes the total rate of proton influx/efflux through all membrane transport mechanisms. This value is negative when protons are leaving the cell and positive when protons are taken-up by the cell. For the case of *C. acetobutylicum*, the SPF is highly negative during exponential growth (acidogenesis) and turns slightly positive during the stationary phase (solventogenesis). In this research, the SPF was constrained to specific values by constraining the proton exchange reaction (the total flux of protons in/out of the systems boundary). The SPF range was between −30 $\frac{mmol\;H^{+}}{hr\cdot gDCW}$ (proton efflux) and 5 $\frac{mmol\;H^{+}}{hr\cdot gDCW}$ (proton influx), and the limits were chosen from experimental observations.

### FBrAtio algorithm

Metabolic engineering *in silico* was enabled through the application of flux ratio constraints. In the FBrAtio method developed in this research, flux ratio constraints were incorporated into the stoichiometric matrix directly, enabling the flux balancing problem to be solved by simple linear programming. Several flux ratios were investigated, and a critical metabolic branch point was identified around the use of the acetyl-CoA metabolite pool. In clostridial metabolism, acetyl-CoA can be used to produce multiple acids, solvents, or macromolecules to produce biomass. The routes taken by acetyl-CoA either regenerate or consume different amounts of ATP and NAD(P)^+^. Ultimately, the balancing of these cofactors determines the production of acids and solvents. The large number of degrees of freedom associated with the genome-scale model of clostridial metabolism allows a large number of acid/solvent production combinations that satisfy cofactor balancing and the overall mass balance, while satisfying the objective functions of the optimization. Thus, flux ratios ultimately reduce the number of degrees of freedom of the system and can be used to define selectivity. The following example demonstrates the application of a flux ratio related to the consumption of acetyl-CoA and the incorporation of this flux ratio into the stoichiometric matrix. As shown in Figure 1, two possibilities for acetyl-CoA are (i) usage by the thiolase enzyme (THL) for conversion to acetoacetyl-CoA and (ii) usage by the phosphotransacetylase enzyme (PTA) for conversion to acetyl phosphate. The reactions catalyzed by these enzymes are given Equations. 1 and 2.

(1) $$2\;acetyl-CoA\overset{THL}{\to }\;acetoacetyl-CoA+CoA$$

(2) $$acetyl-CoA+orthophosphate\overset{PTA}{\to }acetyl-phophate+CoA$$

Next, a ratio of fluxes for these reactions is assumed. For this hypothetical example, it is assumed that twice as much flux proceeds through the THL reaction (Equation 1) than the PTA reaction (Equation 2). Ultimately, biochemical origins of differing fluxes through competing reactions, where known, can be used to calculate flux ratios. The flux through the reaction catalyzed by THL is *f*(*rTHL*) and the flux through the PTA catalyzed reaction is *f*(*rPTA*). This flux ratio is called *f*(*rTHL*):*f*(*rPTA*) and is represented as Equation 3.

(3) $$f\left(rTHL\right):f\left(rPTA\right)=\frac{f\left(rTHL\right)}{f\left(rPTA\right)}=2$$

To build this flux ratio constraint into the stoichiometric matrix (*S*), first Equation 3 is rearranged to the following.

(4) $$f\left(rTHL\right)-2f\left(rPTA\right)=0$$

Next, a new row is added to the stoichiometric matrix. In this new row, two values are added (all other values in the row are zero). In the column representing the reaction catalyzed by THL (Equation 1), the coefficient 1 is added to the matrix. In the column representing the reaction catalyzed by the PTA, the coefficient −2 is added (in the new row). With these additions, when the flux balance equation (*S · v* = 0) is solved, the ratio of fluxes for the reactions of Equation 1 and Equation 2 will be exactly 2. If the flux ratio chosen leads to an impossible solution of the metabolic network, no solution will be found to the flux balance equation.

### Simulations performed

The goal of this research was to develop a method of constraining a metabolic network so that metabolic engineering can be performed *in silico*. Until now, gene knockouts have been the dominant strategy for designing metabolic engineering strategies *in silico*. However, flux ratios offer the ability to include over-expression and flux re-direction at key branch points in a metabolic network. These results can offer a snapshot of the metabolic potential of the engineered cell and offer the metabolic engineer an experimental target to achieve these results. Simulations performed in this research focus on using FBrAtio to reproduce metabolic engineering strategies that have been experimentally validated in *C. acetobutylicum*[6,43,50]. In particular, simulations were performed with the *i*CAC490 model in which the glucose uptake rate and the SPF were the only specified membrane transport fluxes. Next, FBrAtio was applied to achieve the experimentally observed wild-type metabolic activity of *C. acetobutylicum*. The following metabolic characteristics were sought on a qualitative level: (i) at highly negative values of SPF (acidogenesis), acetate and butyrate are produced in high quantities, (ii) high hydrogen production accompanies acidogenesis, (iii) solvents are produced at SPF values close to zero and slightly positive, (iv) hydrogen production decreases during solventogenesis, (v) the maximum growth rate of the culture occurs during acidogenesis, (vi) the production of butyrate is slightly greater than the production of acetate and much greater than the production of lactate, and (vii) the production of butanol is greater than the production of acetone and is much greater than the production of ethanol. Following obtaining a qualitatively accurate simulation of wild-type metabolism, additional flux ratios were applied through FBrAtio in attempt to predict the following experimental observations [43,50]. Knockdown of the CoAT (by asRNA) resulted in increased butanol to acetone selectivity, but this strategy resulted in decreased ethanol and butanol production [50]. The asRNA was designed against the mRNA of the *cftB* gene in particular, which is a part of the tricistronic operon (*aad-ctfA-ctfB*). It was hypothesized that AAD activity was also compromised by this asRNA construct, so *aad* was over-expressed under its native promoter. Significantly higher ethanol and butanol yields were observed as a result of this metabolic engineering strategy [43]. Flux ratios were designed to (i) knockdown CoAT activity only, (ii) knockdown activity of both CoAT and AAD, and (iii) knockdown CoAT while over-expressing AAD at and above wild-type levels.

## Results

### Simulations with a minimal set of constraints

The *i*CAC490 model was simulated with a glucose uptake rate constrained to 10 $\frac{mmol}{hr\cdot gDCW}$ and the SPF was varied between −30 and 5 $\frac{mmol\;H^{+}}{hr\cdot gDCW}$. Only thermodynamic reversibility constraints were used initially. Results showed acidogenic and solventogenic metabolic phases that coincided with SPF values [12]. Results also showed a maximum specific growth rate at an SPF value of −10 $\frac{mmol\;H^{+}}{hr\cdot gDCW}$, which is consistent with previous findings [2,12]. However, during acidogenesis, acetate was the primary acid produced, and acetone was the primary solvent produced during solventogenesis. Hydrogen (H_2_) production was also maximized during solventogenesis. These characteristics are not consistent with experimental observations. Since only acetate was produced in acidogenesis, this demonstrates that the network required the generation of ATP. Since butyrate or ethanol was not produced, this means that NAD^+^ was regenerated in a futile cycle elsewhere in the network. The ability of the network to artificially balance NAD(P)^+^/NAD(P)H also explains why hydrogen production remained high during solventogenesis. By maximizing the specific growth rate of the cell and minimizing the total flux of the system, flux in longer pathways, such as butyrate/butanol production were minimized in favor of shorter ATP (acetate) and NAD^+^ (futile cycle) regenerating pathways.

### Approximating wild-type metabolism with FBrAtio

**Figure 2:**
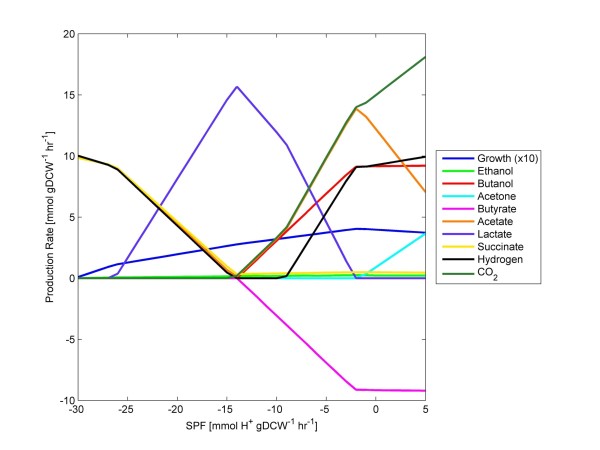
**FBA results of wild-type metabolism using *i*CAC490.** The model was simulated given (i) a glucose uptake rate of 10 $\frac{mmol}{hr\cdot gDCW}$, (ii) varied SPF, and (iii) constraints listed in Table 1. These results do not coincide with experimental observation.

Obvious futile cycles allowing artificial NAD(P)^+^/NAD(P)H balancing were located and corrected, but the problems described above remained. Given the ability of the metabolic network to artificially balance NAD(P)^+^/NAD(P)H without using the acid/solvent production pathways, additional constraints and flux ratio constraints were implemented. First, the reactions involving the ferredoxins were further constrained for irreversibility. These reactions and their updated constraints are given in Table 1. Simulation of metabolism, given these and glucose uptake and SPF constraints are shown in Figure 2. In this simulation, the following results do not coincide with experimental observations: (i) succinate was produced in high levels during acidogenesis, (ii) the production of acetate and lactate far exceed butyrate production, (iii) only butyrate was re-consumed (negative flux values), (iv) the amount of butyrate re-consumed exceeded the amount of butyrate produced, (v) butanol was produced well before acetone, and (vi) hydrogen (H_2_) production fell to zero and rose during solventogenesis. These results show that during early production of butanol (before production of acetone), butyrate was consumed through the production pathway. This is consistent with previous findings [7,58], and the thermodynamic reversibility calculations [54] insist the reactions catalyzed by the butyrate kinase (BK) and the phosphotransbutyrylase (PTB) (see Figure 1) remain reversible.

**Table 1:** Reactions and updated constraints involving the ferredoxins

**Enzyme Name**	**Reaction**	**Lower Bound**	**Upper Bound**
PFO: pyruvate ferredoxin oxidoreductase	$\begin{array}{l} CoA+pyruvate+Fd_{\mathrm{Ox}}\to \\ \;CO_{2}+acetyl-CoA+H^{+}+Fd_{\mathrm{Red}} \end{array}$	0	1000
HYDA: hydrogenase	$2\;H^{+}+Fd_{\mathrm{Red}}\to H_{2}+Fd_{\mathrm{Ox}}$	0	1000
FNO: ferredoxin NAD^+^ oxidoreducatase	$\begin{array}{l} NAD^{+}+H^{+}+Fd_{\mathrm{Red}}\to \\ \;NADH+Fd_{\mathrm{Ox}} \end{array}$	0	1000
FNPO: ferredoxin NADP^+^ oxidoreductase	$\begin{array}{l} NADP^{+}+H^{+}+Fd_{\mathrm{Red}}\to \\ \;NADPH+Fd_{\mathrm{Ox}} \end{array}$	0	1000

*The reduced ferredoxin (Fd_Red_) and the oxidized ferredoxin (Fd_Ox_).

A total of five flux ratio constraints were found necessary to generate simulations of metabolism that were qualitatively consistent with experimental observations. First, the butyrate to acetate uptake ratio was constrained according to previously published findings [8]. The assumption was made that the extracellular butyrate concentration was twice that of the extracellular acetate concentration. From the original relationship [8], this meant that the flux of butyrate uptake through the CoAT, *f*(*rCoAT, butyrate*), to acetate uptake, *f*(*rCoAT, acetate*), was equal to 0.63, as shown in Equation 5. This flux ratio is referred to as *f*(*rCoAT, butyrate*):*f*(*rCoAT, acetate*).

(5) $$\begin{array}{ll} f\left(rCoAT,butyrate\right) & :f\left(rCoAT,acetate\right)\\ & =\frac{f\left(CoAT,butyrate\right)}{f\left(CoAT,acetate\right)}=0.63 \end{array}$$

Implementing this flux ratio alone led to high initial production of succinate, and hydrogen production increased during solventogenesis. Over-production of ethanol and lactate was also observed. So, a flux ratio was installed to direct the conversion of pyruvate to either lactate, through the lactate dehydrogenase (LDH), or to acetyl-CoA through the pyruvate ferredoxin oxidoreductase (PFO) (see Table 1). This flux ratio is shown as Equation 6. This ratio was set equal to 10 to coincide with published experimental observations that lactate production is much less than acetate and butyrate production [2].

(6) $$\frac{f\left(rPFO\right)}{f\left(rLDH\right)}=10$$

Simulations with these two flux ratios were characterized by (i) high acetate and ethanol production, (ii) low butyrate and butanol production, (iii) high initial secretion of succinate, and (iv) low initial production of hydrogen. The secretion of succinate was investigated next. This is indicative of high fluxes through the TCA cycle. No significant succinate export has been reported for *C. acetobutylicum*, so an optimized metabolic model must produce only minimal succinate (if any). In the *i*CAC490 model, production of oxaloacetate from pyruvate requires HCO^-^_3_. When CO_2_ is produced, it is either (i) transported out of the cell or (ii) converted to HCO^-^_3_. The fate of CO_2_ is determined by physicochemical properties of the intracellular environment and has a significant impact on intracellular metabolism. Thus, a ratio constraint for CO_2_ export against conversion was derived. This ratio is shown in Equation 7 and was set equal to 5 to approximate intracellular conditions. This value was chosen because it led to effective simulations. The physicochemical nature of this flux ratio constraint is currently under investigation.

(7) $$\frac{f\left(CO_{2}\;export\right)}{f\left(CO_{2}\;conversion\right)}=5$$

**Figure 3:**
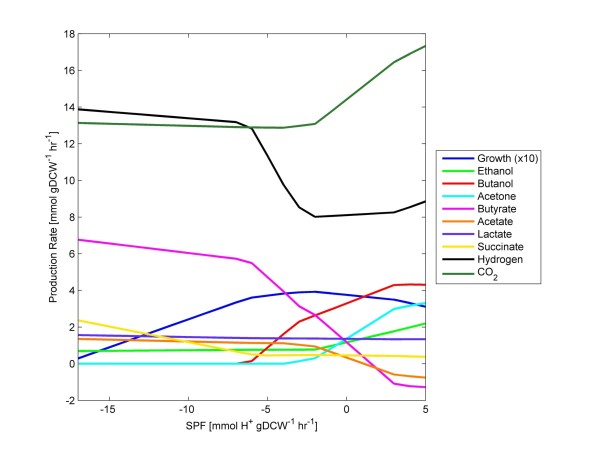
**FBrAtio results of wild-type metabolism using *i*CAC490.** The model was simulated model given (i) a glucose uptake rate of 10 $\frac{mmol}{hr\cdot gDCW}$, (ii) varied SPF, (iii) constraints listed in Table 1, (iv) unidirectional acetate and butyrate secretion by diffusion, and (v) *f* (*rTHL*) *f* (*rPTA*) = 2, (vi) *f* (*rTHL*): *f* (*rAAD*_1) = 10, (vii) *f* (*CO*_*2*_*export*): *f* (*CO*_*2*_*conversion*) = 5, (viii) *f* (*rPFO*): *f* (*rLDH*) = 10, (ix) *f* (*rCoAT, butyrate*):*f* (*rCoAT, acetate*) = 0.63. Results qualitatively fit wild-type metabolism.

This flux ratio constraint corrected hydrogen production (i.e., high hydrogen production during acidogenesis and reduced during solventogenesis) and minimized succinate secretion, which is consistent with experimental observations. This constraint also resulted in increased CO_2_ production during solventogenesis, relative to production during acidogenesis. However, metabolic activity was still characterized by (i) high acetate and ethanol production and (ii) low butyrate and butanol production (results not shown). To address this, flux ratios were constructed around the use of the acetyl-CoA metabolite pool in clostridial metabolism (see Figure 1). Acetyl-CoA can be utilized to (i) produce ATP through acetate production, (ii) regenerate NAD^+^ through ethanol production, or (iii) balance both ATP and NAD^+^ by producing butyrate. However, the shorter metabolic pathways result in acetate and ethanol production and accommodate minimizing the total flux of the metabolic network. To approximate wild-type clostridial metabolism, flux must proceed from acetyl-CoA through the thiolase (THL) with greater flux than through the phosphotransacetylase (PTA) towards acetate or through the bifunctional aldehyde/alcohol dehydrogenase (AAD). To ensure this, additional flux ratio constraints were derived. The ratio of metabolic flux through the THL relative to the PTA, *f*(*rTHL*)*:f*(*rPTA*), was set equal to 2, and the flux through the THL relative to the AAD, *f*(*rTHL*)*:f*(*rAAD_1*), was set equal to 10. This ensured the majority of acetyl-CoA was sent to the butyrate producing pathway while a greater amount of acetyl-CoA was converted to acetate than was converted to ethanol. These flux ratios are shown in Equations 8 and 9. Simulation results are shown in Figure 3. Due to the constant glucose uptake rate, solutions to the flux balance equation were only possible for SPF values greater than −17 $\frac{mmol\;H^{+}}{hr\cdot gDCW}$. In a previous study [12], much higher glucose uptake rates enabled flux solutions at SPF values approaching −55 $\frac{mmol\;H^{+}}{hr\cdot gDCW}$. In the simulations shown in Figure 3, a roughly 1:1 production ratio of H_2_/CO_2_ was observed during exponential growth, and the production of H_2_ decreased in solventogenesis, while the production of CO_2_ increased. This is consistent with experimental observations [59]. Butyrate was produced in greater amounts than acetate, and both acids were taken up during solventogenesis. The production of butanol was greater than that of ethanol and was similar to that of acetone. The production of lactate was minimal, as was the secretion of succinate. The maximum specific growth rate of the culture occurred towards the end of acidogenesis but prior to solventogenesis onset. Thus, when the flux ratio constraints of Equations 59 were applied through FBrAtio, the *i*CAC490 genome-scale model was able to capture the major properties of wild-type metabolism. This was done for the first time without constraining acids and solvents production rates directly using constraints on transporters or exchange fluxes.

(8) $$f\left(rTHL\right):f\left(rPTA\right)=\frac{f\left(rTHL\right)}{f\left(rPTA\right)}=2$$

(9) $$f\left(rTHL\right):f\left(rAAD\_1\right)=\frac{f\left(rTHL\right)}{f\left(rAAD\_1\right)}=10$$

### asRNA knockdown of CoAT only

**Figure 4:**
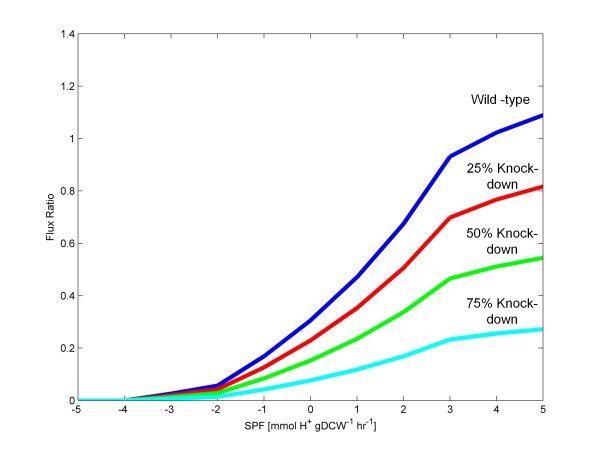
**The wild-type *f*(*rCoAT*):*f*(*rBHBD*) flux ratio.** Knockdowns of the CoAT of (i) 25%, (ii) 50%, and (iii) 75% are shown.

Previous research found that knockdown of the CoA transferase using asRNA technology resulted in a more favorable butanol to acetone selectivity. Although, overall reduced butanol yields were observed [50], possibly due to simultaneous AAD knockdown. To determine if FBrAtio can predict of these findings, a flux ratio constraint was derived to simulate knockdown of the CoAT. It was assumed this knockdown led to a decreased flux of acetate and butyrate re-uptake. As shown in Figure 1, the CoAT converts acetoacetyl-CoA to acetoacetate while transporting acetate or butyrate into the cell and converting them to acetyl-CoA or butyryl-CoA, respectively. Acetoacetyl-CoA, on the other hand, can also be converted to β-hydroxybutyryl-CoA by the β-hydroxybutyryl-CoA dehydrogenase (BHBD). So, a flux ratio constraint was derived to specify the distribution of β-hydroxybutyryl-CoA utilized by the CoAT and the BHBD. This flux ratio is called *f*(*rCoAT*)*: f*(*rBHBD*), where the flux through the CoAT includes fluxes for both acetate and butyrate re-uptake. In the wild-type simulations shown in Figure 3, given an SPF of 5 $\frac{mmol\;H^{+}}{hr\cdot gDCW}$, the flux through the CoAT was 2.03 $\frac{mmol}{hr\cdot gDCW}$ for acetate re-uptake and 1.28 $\frac{mmol}{hr\cdot gDCW}$ for butyrate re-uptake (total CoAT flux of 3.31 $\frac{mmol}{hr\cdot gDCW}$). The wild-type flux through BHBD was 3.04 $\frac{mmol}{hr\cdot gDCW}$ to yield a wild-type *f*(*rCoAT*)*: f*(*rBHBD*) flux ratio of 1.09. The flux ratio is given in Equations 10. However, this flux ratio in the wild-type was a function of the SPF, and this relationship is shown in Figure 4. To simulate knockdown of the CoAT, two separate approaches were attempted. The first approach continued to treat this flux ratio as a function of the SPF. The flux ratio was constrained to values of (i) 75%, (ii) 50%, and (iii) 25% of the wild-type value, as shown in Figure 4. The second approach was to fix the flux ratio to a specified value for all values of the SPF. The flux ratios chosen were: (i) 1, (ii) 0.5, (iii) 0.1, and (iv) 0.01.

(10) $$\frac{f\left(rCoAT\right)}{f\left(rBHBD\right)}=1.09$$

**Figure 5:**
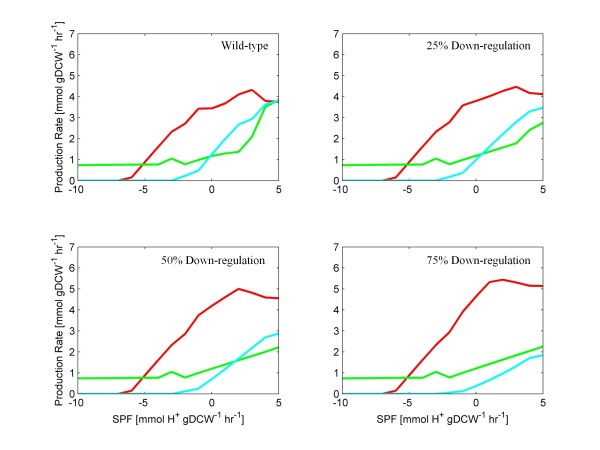
**FBrAtio predictions of solvent production using the *i*CAC490 model and ratios of Figure**4**.** The model was simulated given (i) a glucose uptake rate of $10\frac{mmol}{hr\cdot gDCW}$, (ii) varied SPF, (iii) constraints listed in Table 1, (iv) unidirectional acetate and butyrate secretion by diffusion, (v) *f* (*rTHL*): *f* (*rPTA*) = 2, (vi) *f* (*rTHL*): *f* (*rAAD*_1) = 10, (vii) *f* (*CO*_*2*_*export*): *f* (*CO*_*2*_*conversion*) = 5, (viii) *f* (*rPFO*): *f* (*rLDH*) = 10, (ix) *f* (*rCoAT, butrate*): *f* (*rCoAT, acetate*) = 0.63, and (x) *f* (*rBHBD*) flux ratios defined in Figure 4 as a function of the SPF. The following curves are shown: acetone (cyan), butanol (red), and ethanol (green).

FBrAtio results with the *f*(*rCoAT*)*:f*(*rBHBD*) flux ratios of Figure 4 are shown in Figure 5. In particular, results of acetone, butanol, and ethanol predictions are shown to focus on the impact of CoAT asRNA knockdown on solvent production. The SPF range shown is from −10 to 5 $\frac{mmol\;H^{+}}{hr\cdot gDCW}$. Consistent with published experimental results [43,50], simulated knockdown of the CoAT resulted in an increased butanol to acetone ratio. Significantly lower acetone production (~50% reduction) was predicted with increased butanol production (~25% increase) when CoAT activity was down-regulated by 75%. Significant about these simulation results is that the impact of “fine-tuning” an asRNA construct can be observed through simulations using flux ratio constraints.

**Figure 6:**
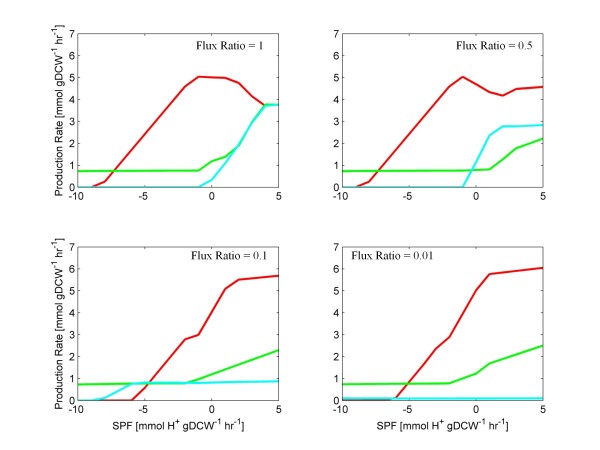
**FBrAtio predictions of solvent production using the *i*CAC490 model and fixed ratios.** The model was simulated given (i) a glucose uptake rate of $10\frac{mmol}{hr\cdot gDCW}$, (ii) varied SPF, (iii) constraints listed in Table 1, (iv) unidirectional acetate and butyrate secretion by diffusion, (v) *f* (*rTHL*): *f* (*rPTA*) = 2, (vi) *f* (*rTHL*): *f* (*rAAD_*1) = 10, (vii) *f* (*CO*_*2*_*export*): *f* (*CO*_*2*_ conversion) = 5, (viii) *f* (*rPFO*): *f* (*rLDH*) = 10, (ix) *f* (*rCoAT, butrate*): *f* (*rCoAT, acetate*) = 0.63, and (x) constant *f* (*rCoAT*): *f* (*rBHBD*) flux ratios of 1, 0.5, 0.1, and 0.01. The following curves are shown: acetone (cyan), butanol (red), and ethanol (green).

It was important to determine whether the *f*(*rCoAT*)*:f*(*rBHBD*) flux ratio must be represented as a function of the SPF (Figure 4). In further simulations, this flux ratio constraint was artificially set and held constant for all values of the SPF. FBrAtio results are shown in Figure 6 for flux ratios of: (i) 1, (ii) 0.5, (iii) 0.1, and (iv) 0.01. Similar trends were obtained. As the *f*(*rCoAT*)*: f*(*rBHBD*) flux ratio decreased, metabolic flux was forced through the BHBD enzyme (rather than through CoAT), resulting in (i) decreased acetate/butyrate re-uptake, (ii) decreased acetone production, and (iii) increased flux through the butanol production pathway. The exaggerated flux ratio simulations of Figure 6 (ratios of 0.1 and 0.01) show the potential of effective asRNA or gene knockout of the CoAT. However, even though CoAT knockdown simulations resulted in predicted phenotypes with improved butanol to acetone selectivity, these phenotypes did not show overall reduced ethanol and butanol production. This suggests AAD knockdown may be required to impact alcohol yields, which is discussed in the next section.

### Knockdown of both CoAT and AAD

**Figure 7:**
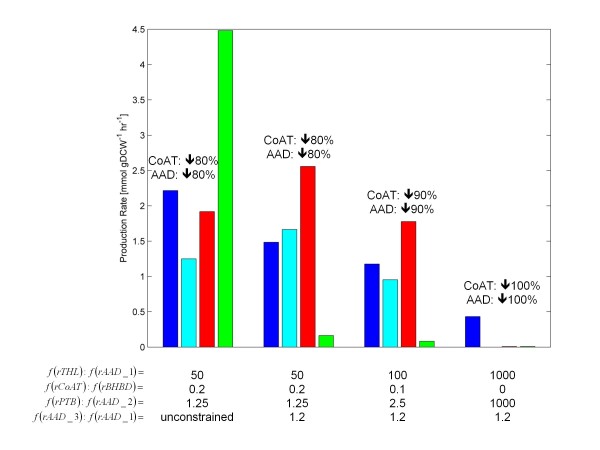
**FBrAtio predictions using the *i*CAC490 model given various levels of CoAT and AAD knockdown.** Shown are predictions of growth (x10) (blue), acetone (cyan), butanol (red), and ethanol (green) during solventogenesis (SPF = 5). The following flux ratio constraints were applied: (i) *f*(*rTHL*):*f*(*rAAD*_1), (ii) *f*(*rCoAT*):*f*(*rBHBD*), (iii) *f*(*rPTB*):*f*(*rAAD*_2), and (iv) *f*(*rAAD_3*):*f*(*rAAD*_1). The following were held constant: (i) glucose uptake rate of $10\frac{mml}{hr\cdot gDCW}$, (ii) constraints listed in Table 1, (iii) *f*(*rTHL*):*f*(*rPTA*) = 2, (iv) *f*(*CO*_*2*_*export*):*f*(*CO*_*2*_*conversion*) = 5, (v) *f*(*rPFO*):*f*(*rLDH*) = 10, and (vi) *f*(*rCoAT, butyrate*):*f*(*rCoAT, acetate*) = 0.63.

In published research [43,50], it was suspected that the *aad* gene was knocked-down when the asRNA for *cftB* (CoAT) was applied. The resulting strain produced very little ethanol or butanol. Since these two genes reside in the same operon, this hypothesis is valid. The previous results (Figures 5 and 6) showed that the butanol to acetone ratio was increased with CoAT knockdown. Simulations of the knockdown of both CoAT and AAD to defined levels using FBrAtio are shown in Figure 7. The AAD was knocked-down by the use of two flux ratio constraints. In these simulations, knockdown of AAD must be accommodated at two metabolic branch points (i) acetyl-CoA and (ii) butyryl-CoA (see Figure 1). To simulate the AAD knockdown, the ratio of flux diverted through AAD, relative to its other choices, was decreased by 80%. This factor was chosen based on the published effectiveness of the originally designed asRNA [50]. To incorporate the knockdown into flux ratio constraints, the *f*(*rTHL*)*:f*(*rAAD_1*) flux ratio constraint was increased from a value of 10 to 50. To implement the flux ratio constraint at butyryl-CoA, a new *f*(*rPTB*)*:f*(*rAAD_2*) flux ratio constraint was created. In wild-type simulations (see Figure 3), this flux ratio was unconstrained and had an average value of 0.52 at an SPF value of 0 $\frac{mmol\;H^{+}}{hr\cdot gDCW}$ and 0 at an SPF value of 5 $\frac{mmol\;H^{+}}{hr\cdot gDCW}$ (assumed value of 0.25 during solventogenesis). At highly negative values of the SPF, this ratio becomes infinite (since no butanol is produced). The *f*(*rPTB*)*:f*(*rAAD_2*) was constrained to 1.25 (80% knockdown over 0.25) over all values of the SPF. The *f*(*rTHL*)*:f*(*rAAD_1*) flux ratio constraint was held constant at 50 (also 80% knockdown). The CoAT was knocked-down 80% by adjusting the *f*(*rCoAT*)*:f*(*rBHBD*) flux ratio to 0.2 over all values of the SPF. Results for solventogenesis (SPF = 5) are shown in Figure 7. Initial simulations revealed a large ethanol production that was not consistent with experimental findings. A closer inspection of genome-wide metabolic fluxes revealed several reactions were involved in futile cycles to produce excess acetaldehyde. To correct this, a new flux ratio constraint was created between AAD_1 and AAD_3 and is shown in Equation 11. This new flux ratio constraint was set to 1.2 since it is known that acetaldehyde is not produced exclusively through AAD_1 (although the exact ratio has not been measured). FBrAtio results given the *f*(*rAAD_3*)*:f*(*rAAD_1*) flux ratio constraint yielded significantly reduced ethanol production. Results with 80% knockdown of CoAT and AAD still showed significant butanol production; however, this was significantly reduced (towards zero) as the percent knockdown was increased.

(11) $$\frac{f(rAAD\_3)}{f(rAAD\_1)}=1.2$$

### Over-express AAD and knockdown CoAT

**Figure 8:**
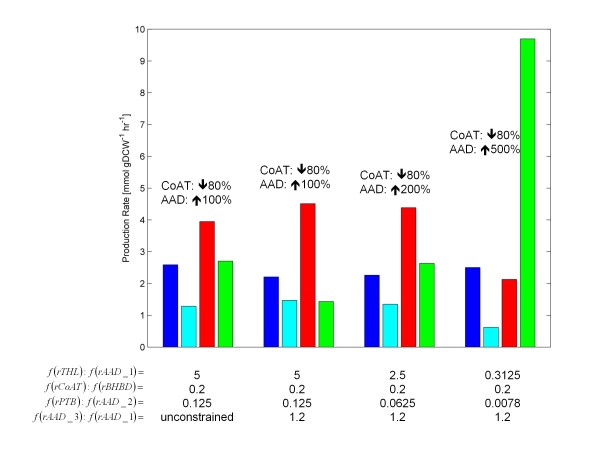
**FBrAtio predictions using the *i*CAC490 model at various levels of CoAT knockdown and AAD over-expression.** Shown are predictions of growth (x10) (blue), acetone (cyan), butanol (red), and ethanol (green) production during solventogenesis (SPF = 5). The following flux ratios were applied: (i) *f*(*rTHL*):*f*(*rAAD*_1), (ii) *f*(*rCoAT*):*f*(*rBHBD*), (iii) *f*(*rPTB*): *f* (*rAAD*_2), and (iv) *f*(*rADD*_3):*f*(*rAAD_*1). The following were held constant: (i) glucose uptake rate of $10\frac{mmol}{hr\cdot gDCW}$, (ii) constraints listed in Table 1, (iii) *f*(*rTHL*):*f*(*rPTA*) = 2, (iv) *f*(*CO*_*2*_*export*):*f*(*CO*_*2*_*conversion*) = 5, (v) *f*(*rPFO*):*f*(*rLDH*) = 10, and (vi) *f*(*rCoAT, butyrate*):*f*(*rCoAT, acetate*) = 0.63.

The AAD was over-expressed using the *f*(*rTHL*)*:f*(*rAAD_1*) and *f*(*rPTB*)*:f*(*rAAD_2*) flux ratio constraints. In this case, the *f*(*rTHL*)*:f*(*rAAD_1*) flux ratio constraint was adjusted from 10 (wild-type) to values of (i) 5, (ii) 2.5, and (iii) 0.3125 to simulate over-expression (over wild-type levels) by (i) 100%, (ii) 200%, and (iii) 500% respectively. The *f*(*rPTB*)*:f*(*rAAD_2*) flux ratio constraint was also adjusted accordingly to simulate these levels of over-expression. FBrAtio results of solventogenesis (SPF = 5) are shown in Figure 8. The over-expression of AAD under control of its native promoter led to large increases in ethanol production in experimental observations [43]. However, in this simulation study, an increase in AAD expression of 500% was required to see this dramatic increase. As AAD over-expression reached 1000% (results not shown), ethanol production was increased an additional 60% (relative to the 500% AAD over-expression level). This result is possibly explained by the presence of multiple copies of the plasmid present in the cell during the experimental trials.

## Discussion

The large number of degrees of freedom in primary clostridial metabolism makes this system challenging to model. Initial efforts [1,7,8] relied on experimentally measured data to fit a basic metabolic model and back-calculate pathways fluxes. With the development of genome-scale models, there was initial enthusiasm that this approach would result in a model capable of predicting the metabolic response of the organism to genetic and environmental manipulations. However, this level of prediction was not achieved by the first genome-scale model for *C. acetobutylicum*[11,12]. This original genome-scale model was updated in this research with additional reactions and thermodynamic constraints. Even with a more complete model and updated constraints, the number of degrees of freedom of the primary metabolic network proved too large to generate meaningful predictions, even of wild-type metabolism. This is evident from the results shown in Figure 2. To build a truly predictive model, care must be taken when determining how proper constraints are imposed. It is important that these constraints not only lead to accurate representations of metabolism but can be manipulated to mimic genetic and environmental perturbations. For example, a common method is to artificially constrain the glucose uptake rate (as was done in this research). From there, constraints can be imposed on product (e.g., acetate, butyrate, butanol, etc.) secretion fluxes to mimic the wild-type metabolism. This approach is detrimental to metabolic engineering. For example, if constraints are placed on secretion of the end-products, how do these constraints change when a genetic manipulation is made elsewhere in the metabolic network (e.g., at the thiolase enzyme)? There is no clear mathematical relationship between a secretion flux constraint and the metabolic flux through an enzyme elsewhere in the network. Thus, constraints that are imposed to achieve accurate representations of metabolism must be imposed at the metabolic engineering targets themselves. However, this leads to the questions, what is a metabolic engineering target? And, how can constraints be imposed there? This research has focused on “branch points” (or critical nodes) of the metabolic network as potential sites of metabolic engineering. The use of acetyl-CoA in clostridial metabolism is a good example of a metabolic branch point. Acetyl-CoA can be used in the production of (i) acetate, (ii) butyrate/butanol, (iii) ethanol, and (iv) macromolecules required for cell growth. Each of these routes produces/consumes different cofactors, and the balancing of these cofactors ultimately determines the cellular phenotype.

The use of metabolic flux ratio constraints through FBrAtio enabled qualitatively accurate modeling of acidogenic and solventogenic metabolism of *C. acetobutylicum* using the new *i*CAC490 genome-scale model. The use of flux ratios allows for constraints to be placed directly at points where metabolic engineering strategies can be applied. For example, flux ratios can be manipulated to achieve a desired result (e.g., maximized butanol production). Then, genetic manipulations such as (i) over-expression, (ii) knockout, and (iii) asRNA knockdown can be applied to achieve the optimum ratios. In this research, flux ratio constraints were implemented to achieve a qualitative picture of metabolism that mimics experimental observations. As a proof of concept, the wild-type and two engineered strains analyzed were consistent with published experimental results. The case of AAD over-expression went a step further and exposed a possible metabolic engineering limit to re-routing flux into the alcohol production pathways. This suggests that the approach of flux ratio constraints is tunable. The flux values obtained here were not converted into concentrations of metabolites and biomass and compared directly to published values. The results obtained here are qualitative (not quantitative) pictures of metabolism. There are several reasons for this. First, a fixed glucose uptake rate of 10 $\frac{mmol}{hr\cdot gDCW}$ was used for all values of the SPF examined. Previous results [12] have shown that the glucose uptake rate varies with the SPF. However, the relationship between the glucose uptake rate and the SPF remains uncharacterized. At best, a causal relationship can be established between these two with the current level of knowledge. Next, a single biomass equation was used for all values of the SPF examined. Previous research has shown that the biomass composition, including the maintenance ATP requirement, of *C. acetobutylicum* changes with the SPF [10,12]. To obtain quantitatively accurate predictions, one must first understand the relationships that exist between glucose uptake and biomass composition with the SPF. While research is underway to uncover these relationships, the use of parameters associated with exponential growth seemed to be sufficient with the FBrAtio approach.

FBrAtio is a new method to derive metabolic engineering strategies to achieve optimum phenotypes. The concept of using metabolic flux ratios was initially developed with the METAFoR approach [45,47]. It enabled researchers to determine how multiple biosynthetic pathways contributed to the production of a metabolite pool. This enabled identification of new metabolic pathways and regulatory mechanisms. Since the implementation of FBrAtio accommodates the use of linear programming, flux ratios found with METAFoR can now easily be applied to appropriate genome-scale models using the techniques described in the Methods section (see Equations 14). The FBrAtio approach is different from METAFoR in that it considers how a metabolite pool is distributed as a substrate among competing enzymes. Of course, this process is governed by thermodynamics. This means that enzyme availability and intermediate accumulation downstream (among other factors) are responsible for flux ratios in physical systems. The FBrAtio approach can lead a metabolic engineer to optimum flux ratios, and enzyme availability can be manipulated through gene (i) over-expression, (ii) knockout, or (iii) partial knockdown. However, the FBrAtio approach cannot predict the potential accumulation of downstream intermediates once flux is redirected. This remains a problem for the experimentalist that may be addressed through additional gene over-expression or enzyme engineering.

The FBrAtio approach is presented in detail here and is applied to model previously published metabolic engineering approaches in *C. acetobutylicum*. Obviously, the full potential of FBrAtio will be realized when it can be used systematically. To do this, algorithms are needed to identify critical nodes (metabolite pools) in the metabolic network where flux ratios can be optimized to produce a desired phenotype. Research is currently underway to address this challenging task. The end result will provide the metabolic engineer with a list of flux ratios that can be manipulated using existing toolsets. Although additional complications may be encountered in some cases due to unforeseen regulatory interactions, the FBrAtio approach has the potential to provide effective “fine-tuned” metabolic engineering strategies.

## Conclusions

The FBrAtio approach for -incorporating metabolic flux ratio constraints into a genome-scale metabolic network and generating solutions using simple linear programming was developed in this research. The approach proved effective in modeling wild-type metabolism of *C. acetobutylicum.* FBrAtio was then applied to metabolically engineered strains, and a high ethanol producing strain was effectively modeled. A nonlinear relationship exists between the flux ratios at a critical node and the resulting phenotype. FBrAtio is capable of capturing these nonlinearities. How flux ratio constraints can be used to design metabolic engineering strategies is currently a subject of much future research, and the developments presented here represent the first steps toward truly predictive genome-scale models that can accurately reflect the impacts of genetic and environmental manipulations.

## **Competing interests**

The authors declare that they have no competing interests.

## Authors’ contributions

MM is responsible for constructing the new genome-scale model of *C. acetobutylicum,* initially developing the FBrAtio calculations, and writing initial sections of the manuscript. JY also assisted with developing the FBrAtio approach and assisted in compiling the genome-scale model. BF made significant contributions to the background section of the manuscript and assisted in editing. RS conceived the idea of the FBrAtio approach and contributed significant writing and editing to the manuscript. All authors read and approved the final manuscript.

## Supplementary Material

Additional file 1 ***i*CAC490_Spreadsheet.** Click here for file

Additional file 2 ***i*CAC490_SBML.** Click here for file
